# Radiation Recall Phenomenon Following Vaccination in a Patient With Prior Radiation to Lower Extremity Liposarcoma

**DOI:** 10.7759/cureus.84907

**Published:** 2025-05-27

**Authors:** Anna Chung, Asal Rahimi, Narine Wandrey, Mona Arbab

**Affiliations:** 1 Medicine, University of Texas Southwestern Medical Center, Dallas, USA; 2 Radiation Oncology, University of Texas Southwestern Medical Center, Dallas, USA

**Keywords:** post-radiation immune response, pro-inflammatory cytokines, radiation induced bullous pemphigoid, radiation-induced tissue damage, radiation recall phenomenon, skin and mucosal toxicity, vaccination-induced inflammation

## Abstract

Radiation recall phenomenon (RRP) is a rare inflammatory reaction in previously irradiated tissue triggered by agents such as chemotherapy or vaccines. A 67-year-old female patient who had undergone radiation to the right thigh for liposarcoma developed a blister at the same site during whole breast radiation for invasive lobular carcinoma. This occurred three days after receiving COVID-19, respiratory syncytial virus (RSV), and influenza vaccines. The reaction progressed to an ulcer over two months, while her active breast radiation site remained unaffected. This case highlights vaccination as a potential RRP trigger and the selective involvement of prior radiation fields. Awareness of RRP in vaccinated patients with a history of radiation is essential, warranting further research into its mechanisms.

## Introduction

Radiation recall phenomenon (RRP) or radiation recall dermatitis (RDD) is a rare inflammatory skin reaction occurring in a previously irradiated area following exposure to triggers like chemotherapy, vaccination, antibiotics, or iodine [1-3]. It is characterized by a localized skin reaction that mimics acute radiation dermatitis, even though the triggering agent is administered long after the completion of radiation therapy. The pathophysiology of RRP remains uncertain, but several mechanistic theories have been proposed to explain this phenomenon [1]. 

The leading hypotheses for RRP pathogenesis include stem cell depletion in irradiated tissues leading to diminished regenerative capacity, vascular damage causing localized drug accumulation, epithelial stem cell sensitivity or genetic mutations induced by radiation, and idiosyncratic drug hypersensitivity reactions [4]. Radiation may induce stable changes in cells through altered gene expression patterns and cytokine production pathways, priming them for enhanced inflammatory responses upon subsequent exposure to certain agents. These proinflammatory cytokines and cellular adhesion molecules may remain dormant until reactivated by a triggering agent, explaining how the recall effect can occur years after radiation exposure [5]. 

A prospective study on RRP found an overall frequency of 8.8% among patients receiving anticancer drugs after radiotherapy, with the highest incidence occurring when the interval between radiation and drug administration was less than two months [6]. However, the true incidence may be underreported due to variable clinical presentations and lack of standardized reporting systems. Certain chemotherapeutic agents have been more frequently implicated, with taxanes (particularly docetaxel) [7-9], anthracyclines [10-12], and gemcitabine [13-15] showing particularly high associations with RRP. The reaction typically manifests within days to weeks of the triggering exposure, though cases have been reported with intervals ranging from hours to years. 

While RRP is well-documented with certain chemotherapy drugs, the phenomenon has rarely been associated with vaccines. The introduction of COVID-19 vaccines, particularly mRNA vaccines such as those produced by Moderna and Pfizer-BioNTech, has brought about an increase in reports of various cutaneous reactions [2,16]. However, the potential of these vaccines to trigger RRP remains largely unexplored. 

In this paper, we report a unique case of RRP in a patient with a history of sarcoma treatment, who developed a maculopapular rash and a draining ulcer in a previously irradiated site following the administration of Moderna's COVID-19, respiratory syncytial virus (RSV), and influenza vaccines while receiving breast radiation. This case highlights the possibility of vaccine-induced RRP and underscores the importance of clinician awareness as widespread vaccination continues. Understanding this phenomenon is critical for ensuring appropriate monitoring and management in patients with a history of radiation therapy. 

## Case presentation

**Figure 1:**
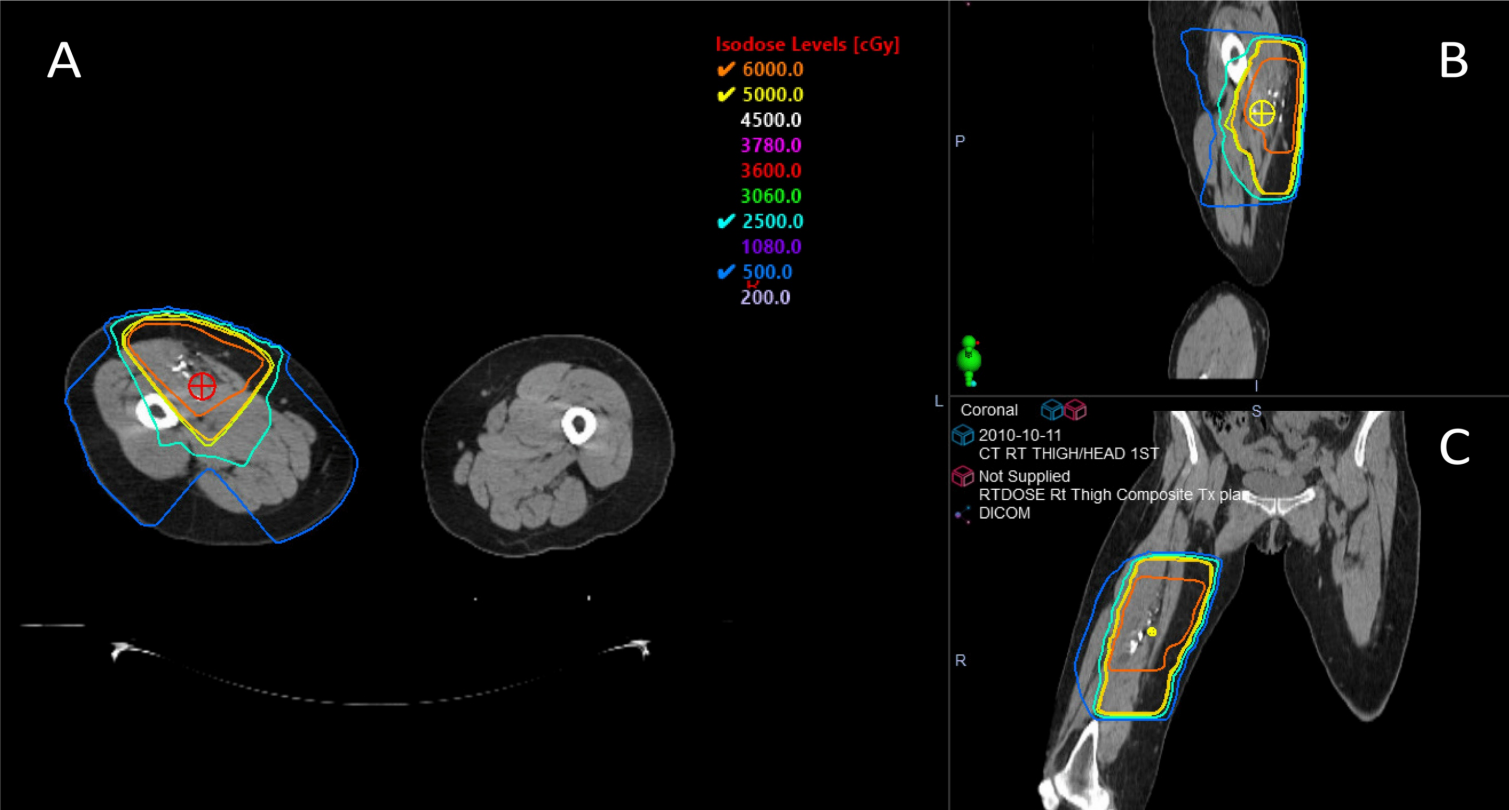
Axial (A), sagittal (B), and coronal (C) views of the right thigh along with the treatment plans for the sarcoma (2010)

A 67-year-old female patient with a medical history of low-grade liposarcoma of the right thigh underwent resection in 2009, followed by a local recurrence treated with a second resection in 2010 and adjuvant radiation therapy (50 Gy in 25 fractions, plus a 10 Gy boost, Figure 1).

In 2021, she presented with another local recurrence of dedifferentiated liposarcoma in the right thigh, leading to further surgical resection without adjuvant treatment. The pathology revealed dedifferentiated liposarcoma, pT4N0, grade 3. 

During surveillance, a chest CT scan revealed a prominent axillary node, leading to a mammogram that identified a 7 mm invasive lobular carcinoma (ILC) of the right breast, grade 2, Estrogen Receptor/Progesterone Receptor (ER/PR) positive, Human Epidermal Growth Factor Receptor 2 (HER2) negative, with Kiel 67 (Ki-67) of 20% (cT1bN0M0). She subsequently underwent a right lumpectomy and sentinel lymph node biopsy, showing pT1bN0, ILC with negative margins. 

**Figure 2:**
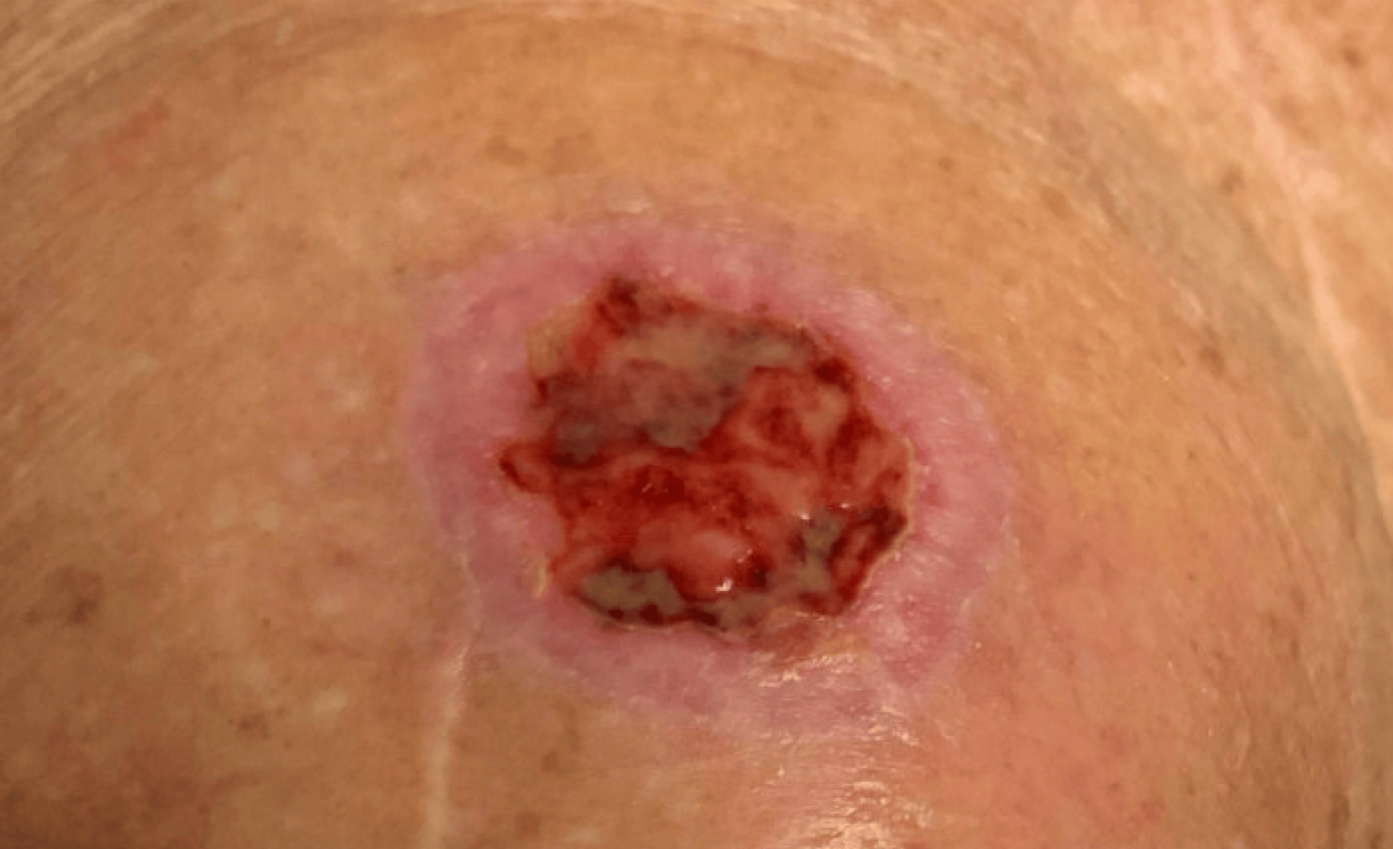
Blister with serosanguinous drainage on the right thigh following breast radiation and COVID-19, RSV, and influenza vaccinations in 2023 RSV: Respiratory syncytial virus

The patient received hypo-fractionated whole-breast radiation (40.05 Gy in 15 fractions, followed by a 10 Gy boost). The boost was administered due to the improved local control in patients under the age of 70 [17]. The patient did not receive any systemic chemotherapy during the observed period, nor any therapeutic drugs known to cause RRP. On her 12th fraction, she developed a blister on her right thigh, associated with serosanguinous drainage (Figure 2).

**Figure 3:**
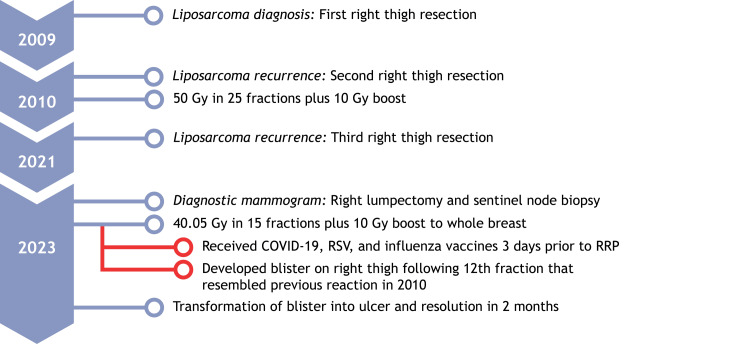
Timeline of liposarcoma and breast cancer treatments with subsequent RRP reaction RRP: Radiation recall phenomenon

This blister resembled a reaction during her earlier sarcoma radiation in 2010. Investigation revealed that she had received Moderna's COVID-19, RSV, and influenza vaccines three days earlier (Figure 3).

The blister transformed into an ulcer over the next few days. The ulcer base contained pink granulation, slough, and necrotic or devitalized tissue with exposed subcutaneous tissue and no signs of infection. The ulcer resolved with wound care after two months. Importantly, the patient’s pre-existing grade 1 radiation dermatitis in the breast did not worsen after vaccination. 

## Discussion

RRP is a rare and unpredictable reaction characterized by the reactivation of inflammatory responses in previously irradiated tissues upon exposure to a triggering agent. In this case, the patient exhibited a maculopapular rash in the irradiated regions and a persistent serosanguinous draining ulcer in an area treated with radiation a decade earlier. Interestingly, the patient’s radiation-induced grade 1 dermatitis in the breast did not worsen following vaccination, suggesting that the phenomenon may exhibit a differential response based on the timing of radiation therapy and individual tissue susceptibility. 

While chemotherapeutic agents remain the most commonly reported triggers for RRP, vaccine-induced cases have emerged in recent literature. Soyfer et al. reported two cases of RRP following COVID-19 vaccination in patients previously treated for soft tissue sarcomas, with manifestations occurring within six days of vaccination [2]. In one case, a painful, burning, erythematous, and mildly exfoliated skin reaction occurred within the radiation field six months after the initial radiotherapy and five days after a COVID-19 vaccine booster. The RRP resolved within days and was treated symptomatically with topical steroids and painkillers. In the second case, a pruritic and erythematous skin reaction developed within the radiation field approximately three weeks after the completion of radiotherapy and six days after a COVID-19 vaccine booster. The RRP resolved within a week with no local therapy or painkillers. 

Similar to our case, these patients developed a reaction exclusively in the previously irradiated area, with the manifestation occurring shortly after vaccination. The reactions emerged within six days after vaccination, comparable to our patient who developed symptoms three days post-vaccination. This temporal relationship strongly suggests causality between vaccination and RRP, particularly with mRNA vaccines, which are known to induce robust immune responses. The simultaneous administration of multiple vaccines may have produced a stronger inflammatory trigger than a single vaccine, potentially explaining the severe ulcerative reaction in our case. This case adds to the growing literature on vaccine-triggered RRP and highlights the potential for multiple simultaneous vaccinations to serve as potent RRP triggers. 

Our case is unique in that the RRP occurred a decade after the initial radiation treatment, suggesting a long-lasting tissue vulnerability to RRP. The selective involvement of the previously irradiated thigh area with sparing of the active breast radiation site aligns with observations by Azria et al., who noted that RRP tends to occur more frequently in areas that received higher radiation doses or underwent multiple courses of radiation, with ulceration as a potential manifestation [4,18]. 

Camidge and Price proposed that RRP might result from stem cell depletion in previously irradiated tissues, leading to diminished healing capacity when exposed to triggering agents [1]. They hypothesized that radiation induces stable changes in cells, including altered gene expression and cytokine production, priming them for enhanced inflammatory responses upon subsequent exposure to various agents. This could potentially explain why our patient’s thigh, which received both the initial treatment (50 Gy) and a boost (10 Gy) a decade earlier, was more susceptible than the breast area undergoing active radiation. The long latency period of 10 years in our case may support Camidge and Price’s theory that radiation-induced changes persist indefinitely. 

The development of a blister progressing to an ulcer in our patient shares characteristics with bullous pemphigoid reactions reported following radiation therapy. Kluger et al. described a case of radiation-induced bullous pemphigoid that developed within a month after radiation therapy for ductal adenocarcinoma [19]. The patient presented with tense blisters confined to the irradiated area, a clinical feature also observed initially in our case. Unlike our case, this patient also required systemic corticosteroid therapy for resolution, and direct immunofluorescence confirmed the diagnosis by revealing linear deposits of IgG and complement component 3 (C3) along the basement membrane. The authors suggested that radiation might uncover basement membrane antigens, triggering an autoimmune response. 

A systematic review of radiation-induced bullous pemphigoid cases identified 21 reported cases in the literature, with latency periods ranging from weeks to 16 years after radiation exposure [20]. This supports the possibility of long-term susceptibility to inflammatory reactions in irradiated tissues, as observed in our patient who developed a blister a decade after radiation therapy. The persistent serosanguinous drainage observed in our patient resembles the clinical features of bullous pemphigoid, although characteristic immunological confirmation was not performed. Most cases of radiation-induced bullous pemphigoid (89%) remained confined to the irradiated field [20], similar to our patient’s localized presentation; however, cases usually require systemic corticosteroid treatment, while our patient’s condition resolved with wound care alone. 

Ben Rejeb et al. described a challenging case that raised similar questions of the nuances between radiation-induced bullous pemphigoid and RRP [21]. The patient in that case developed bullous lesions during her breast cancer treatment initially within the irradiated area, which then spread to other skin areas two months after the completion of radiotherapy with adjuvant trastuzumab. The authors highlight that while both conditions can present with blistering eruptions in irradiated fields, RRP typically requires a specific triggering agent administered after a latency following completion of radiation, whereas radiation-induced bullous pemphigoid represents an autoimmune process that may or may not have a clear secondary trigger. Our case aligns more with RRP than with radiation-induced bullous pemphigoid due to its temporal relationship to vaccination, self-limited course, and lack of immunological features requiring immunosuppressive management. 

This RRP case is unique due to the concurrent administration of multiple vaccines (COVID-19, RSV, and influenza) and the selective involvement of the older thigh radiation field while the active breast radiation site remained unaffected. This selective pattern suggests that established tissue changes from the previous radiation may play a more significant role in RRP susceptibility than acute radiation effects.

The occurrence of RRP following vaccination in our patient offers important insights for counseling future patients with radiation history. While our case does not provide definitive evidence to recommend delaying radiation treatment following vaccination, since RRP occurred only in the previously irradiated field while sparing the active treatment site, clinicians should consider several practical approaches. Patients with prior radiation therapy, particularly those who received high doses (>50 Gy) or multiple radiation courses, should be informed about the small but potential risk of RRP following vaccination, enabling them to seek prompt medical attention if cutaneous reactions develop in previously irradiated areas. When clinically appropriate and not contraindicated by urgent vaccination needs, staggering multiple vaccines rather than administering them simultaneously might reduce the intensity of the immune response potentially triggering RRP. Regarding radiation timing, there appears to be little rationale for delaying treatment based on recent vaccination, though monitoring previously irradiated sites for one to two weeks following vaccination is advisable. Decisions should be individualized based on the urgency of vaccination, the patient's cancer treatment timeline, the extensiveness of the radiation fields, and prior history of radiation-induced skin reactions. For patients requiring vaccination during or shortly after radiotherapy, increased attention to skin care in radiation fields may be warranted, including gentle cleansing and moisturizing practices. It is important to emphasize that the benefits of timely vaccination generally outweigh the rare risk of RRP in patients with cancer, and thus vaccination should not be unnecessarily delayed but rather administered with appropriate counseling and monitoring. Future prospective studies evaluating the incidence of vaccine-induced RRP and identifying potential risk factors would be valuable in refining these recommendations and optimizing care for oncology patients requiring both radiation therapy and vaccinations. 

## Conclusions

This case illustrates that vaccines can trigger RRP, a phenomenon typically associated with chemotherapeutic agents. RRP was limited to the older radiation site, while the breast field showed no exacerbation. RRP occurs in the more intensely treated areas following several radiation courses. Reports exist of bullous pemphigoid reactions years after radiation. With rising COVID-19 vaccination rates, clinicians should be aware of the possibility of RRP, especially in patients with a history of radiation therapy. Early diagnosis and management of RRP can minimize complications and improve outcomes. Further research is needed to explore the mechanisms behind RRP and its variability.
